# Automatic real-time gait event detection in children using deep neural networks

**DOI:** 10.1371/journal.pone.0211466

**Published:** 2019-01-31

**Authors:** Łukasz Kidziński, Scott Delp, Michael Schwartz

**Affiliations:** 1 Stanford University Department of Bioengineering, Stanford, CA, United States of America; 2 Stanford University Department of Mechanical Engineering, Stanford, CA, United States of America; 3 Gillette Children’s Specialty Healthcare, St. Paul, MN, United States of America; 4 University of Minnesota Department of Orthopaedic Surgery, Minneapolis, MN, United States of America; 5 University of Minnesota Department of Biomedical Engineering, Minneapolis, MN, United States of America; The Ohio State University, UNITED STATES

## Abstract

Annotation of foot-contact and foot-off events is the initial step in post-processing for most quantitative gait analysis workflows. If clean force plate strikes are present, the events can be automatically detected. Otherwise, annotation of gait events is performed manually, since reliable automatic tools are not available. Automatic annotation methods have been proposed for normal gait, but are usually based on heuristics of the coordinates and velocities of motion capture markers placed on the feet. These heuristics do not generalize to pathological gait due to greater variability in kinematics and anatomy of patients, as well as the presence of assistive devices. In this paper, we use a data-driven approach to predict foot-contact and foot-off events from kinematic and marker time series in children with normal and pathological gait. Through analysis of 9092 gait cycle measurements we build a predictive model using Long Short-Term Memory (LSTM) artificial neural networks. The best-performing model identifies foot-contact and foot-off events with an average error of 10 and 13 milliseconds respectively, outperforming popular heuristic-based approaches. We conclude that the accuracy of our approach is sufficient for most clinical and research applications in the pediatric population. Moreover, the LSTM architecture enables real-time predictions, enabling applications for real-time control of active assistive devices, orthoses, or prostheses. We provide the model, usage examples, and the training code in an open-source package.

## 1 Introduction

One of the key elements in analysis of gait is the quantitative assessment of gait parameters collected in a reproducible setting. Modern gait laboratories are equipped with motion capture systems that allow experimenters to track trajectories of markers positioned on a subject’s body. After collecting such data, experimenters fit a musculoskeletal model with associated markers and reconstruct body movement. This procedure allows computation of joint angles over time using inverse kinematics. These data are used in a variety of applications, ranging from basic scientific studies about the nature of human movement to clinical assessments used for planning treatments and assessing outcomes.

To make quantitative comparisons, it is conventional to align gait data to predefined landmarks in the gait cycle. These landmarks allow segmentation of the time series into comparable gait cycles and sub-cycles. The standard and well-established parameters of alignment are the foot-contact event (the instant in time when the foot contacts the floor) and the foot-off event (the instant in time when the foot leaves the floor). Whenever the gait data include unambiguous measurement of ground reaction forces, these events can be automatically defined. However, clean force plate data is often missing, especially when individuals with orthopaedic or neurological impairments are being tested [1, 2].

Lacking ground reaction forces, detection can be done automatically in the case of normal gait using basic functions of the markers, such as the velocity of the heel marker or the distance between the heel and the pelvis [3]. In practice, most of the methods have been only validated for normal gait and automatic detection in clinics is uncommon.

In pathological gait, signals from force plates can be corrupted due to partial contact of the foot with the force plate, the presence of the contralateral foot on the force plate, short step length or foot drop, or the presence of assistive devices, such as walkers. For example, vertical acceleration has been shown to be an inappropriate indicator of events in toe walking [4]. Moreover, not all clinics are equipped with force plates, and pathological kinematics, especially of the foot and ankle, introduce significant problems for automatic techniques. Consequently, researchers have sought alternatives to force-plate data using inertial measurement units [5–7], and have developed event detection algorithms appropriate for these systems [8–13]. These solutions require additional hardware, extra time for placing the hardware on the subject, and additional analysis.

**Fig 1:**
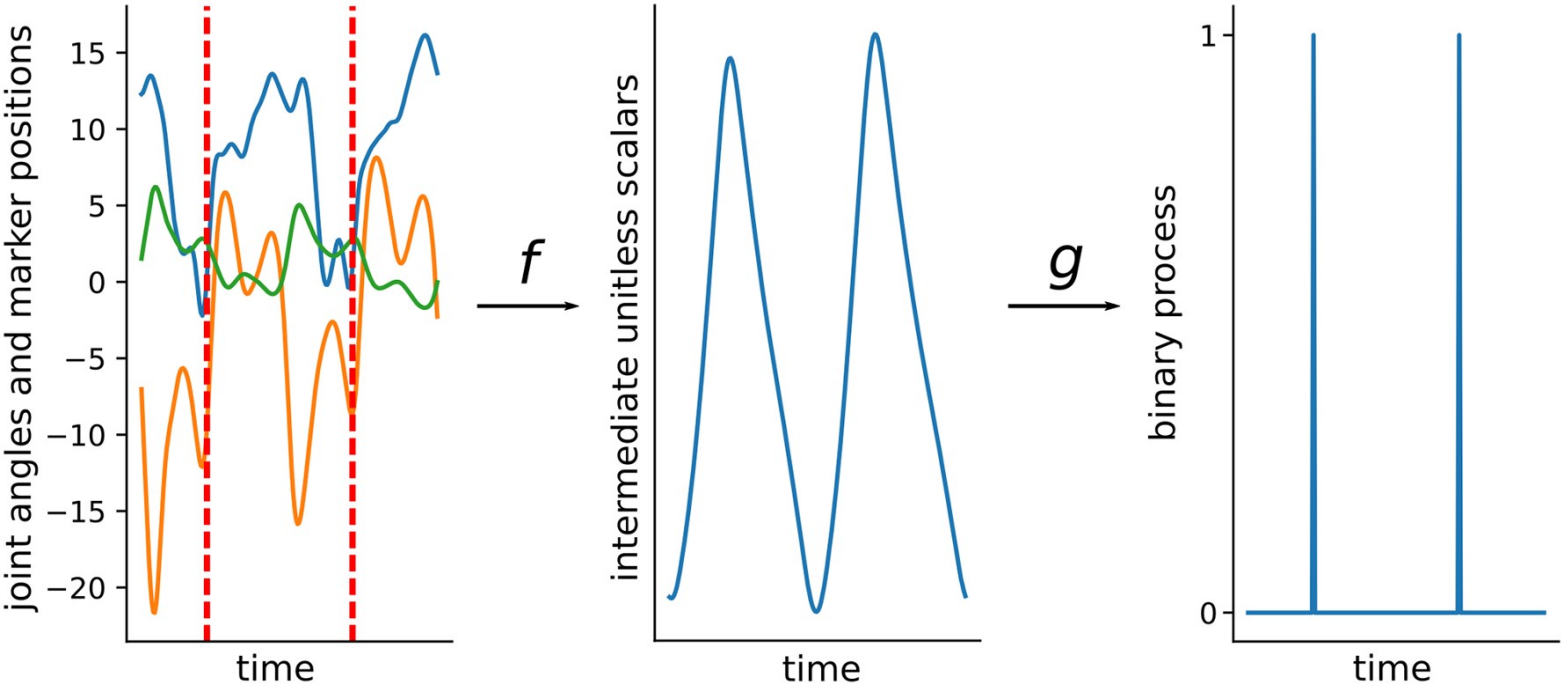
Most of the available algorithms can be decomposed into two steps. In the first step, a multivariate time series of the kinematics and marker trajectories (left plot, with only three variables displayed) is mapped to a univariate time series (middle plot) with some function *f*. In the second step a peak detection algorithm *g* is applied to convert univariate time series into a binary time series (0 when no event and 1 otherwise).

In practice, events in the pathological gait are annotated manually from the kinematic data collected with a motion capture systems. This motivates the search for algorithms automating the time-consuming annotation. Most available algorithms have a similar structure (Fig 1), and can be divided into three categories: *Coordinate-based*, *Velocity-based*, and *Probabalistic*. *Coordinate-based* methods [14, 15] exploit distances between body parts. *Velocity-based* methods [9, 14, 16–20] use velocities of markers. *Probabilistic* methods [21–23] use parametric probabilistic models with manual or data-driven parameter tuning. These algorithms are not widely adopted, rely on heuristics designed for normal or treadmill gait, and commonly fail in the situations encountered in clinics.

In this paper we describe an event detection algorithm that does not require force plates and is applicable to a wide range of pathological gait patterns. The algorithm combines elements of *coordinate-based*, *velocity-based*, and *probabilistic* approaches through a data-driven model. We create a time series model, using coordinate- and velocity-based features to predict the likelihood of an event in a series of frames. To that end, we use a dataset of over 9092 motion capture trials of the gait of children seen in the Center for Gait and Motion Analysis at Gillette Children’s Specialty Healthcare.

There are potential clinical, research related, and economic benefits of automatic gait event detection. From the economic perspective, methods of event detection which only use marker trajectories can reduce the cost of human annotator or additional hardware such as accelerometer or force plates. From the clinical perspective, automatic annotation provides more consistent data and reduces the variance of individual annotators as well as between annotators. From the research perspective, the ability to accurately and efficiently extract multiple gait cycles from a trial reduces the time for annotation and allows the identification of more gait cycles, generating more data for further analysis.

We are releasing the model in order to stimulate further research and enable practical use. The ready-to-use software is published as a GitHub repository (https://github.com/kidzik/event-detector).

## 2 Methods

### 2.1 Data

The participants comprising the dataset were patients visiting Gillette Children’s Specialty Healthcare Center for Gait and Motion Analysis between 1994 and 2017. The participants ranged in age from 4 to 19 years. The dataset contains 18153 trials and 9092 of them have annotated gait events (see Table 1 for details). The patient population is comprised of 73% individuals with a diagnosis of cerebral palsy. The remaining 27% are a mix of neurological (e.g. myelomeningocele), orthopaedic (e.g. patellofemoral syndrome), developmental (e.g. idiopathic toe walking and idiopathic torsion), and genetic disorders.

**Table 1:** Distributions of features of patients in the dataset.

	training set	test set
age (years)	11.4 (sd = 6.2)	11.0 (sd = 4.5)
weight (kg)	35.7 (sd = 17.7)	35.9 (sd = 16.7)
height (cm)	135.7 (sd = 21.6)	135.6 (sd = 21.4)
leg length (cm)	70.3 (sd = 14.0)	70.6 (sd = 12.8)
walking speed (m/s)	0.84 (sd = 0.28)	0.85 (sd = 0.29)

Patients were asked to walk X approximately 15 meters. Each leg is treated separately, yielding 18184 observations with annotations. For every observation the available data consists of trajectories of markers placed on the foot, shank, thigh, and pelvis and rotations of the pelvis, hip, knee, ankle and foot, expressed as Euler angles computed using the plug-in-gait biomechanical model [24, 25]. We refer to these sequences as *kinematic time series*.

Estimation of the joint angles was performed by tracking the markers attached to body, defining segmental coordinate systems, and computing rotations between segments. Data was collected at 120Hz with a VICON system.

During each patient’s visit, multiple trials were collected. The trials were processed for clinical use by trained technicians. In general, a single stride was identified from each trial, delineated by foot-contact and foot-off events for both legs.

The study was approved by the University of Minnesota Institutional Review Board (IRB) and it was granted a waiver. Patients, and guardians, where appropriate, gave informed written consent at the clinical visit for their data to be included. In accordance with IRB guidelines, all patient data was deidentified prior to any analysis.

### 2.2 Data considerations

We designed the algorithm taking into account potential variability in the motion capture protocols. We assume that most of the clinics have a kinematic model producing similar output for joint angles, so we use many of these signals, but we reduce the set of marker trajectory signals to only a few of the most commonly used markers.

Although the input data is close to data used in existing algorithms the advantage of our approach comes from the data-driven nature of the algorithm; instead of deriving heuristics related to particular markers or joint angles, we build a statistical model that finds a relation between gait events and multivariate time series of marker and kinematic data.

### 2.3 Data preprocessing

For each leg *r* ∈ {*left*, *right*} we track observations of five bodies: hip, knee, ankle, toes, and pelvis. In each frame positions of hip, knee, ankle, and toes are stored relative to the pelvis in the same frame. Positions of the pelvis are stored relative to the pelvis in the first frame. In each trial we sample *n*_*r*_ vectors composed of: 3-dimensional position of each body, 3-dimensional velocities, and 3-dimensional acceleration of the pelvis. Input observations are stored as matrices $X_{r}\in R^{n_{r}\times 33}$.

We annotate foot-contact and foot-off events and we treat these manual annotations as the ground truth. For clarity, here we focus on the foot-contact event, the analysis of foot-off event is analogous. For each frame we encode a gait event as 1 and a non-event as 0. As a result, each trial consisting of *n*_*r*_ frames is encoded as a vector of *n*_*r*_ binary values, i.e. $Y_{r}\in {\left\{0,1\right\}}^{n_{r}}$. Our task can now be defined as a prediction of *Y*_*r*_ vector from the observed *X*_*r*_. Formally we are looking for a mapping function *f* such that ${\hat{Y}}_{r}=f\left(X_{r}\right)$, and we want to optimize the mapping to have the prediction ${\hat{Y}}_{r,t}$ as close as possible to the observe data *Y*_*r*,*t*_ for *t* ∈ {1, …, *T*_*r*_}.

### 2.4 Sequence to sequence mapping

We use the framework of sequence to sequence mapping [26]. In this framework, we are finding a function between one time series and another as represented by function *f* in Fig 1. To approximate the function we use a Long Short-Term Memory Network (LSTM) [27] due to its high flexibility. Thanks to the large size of our dataset, we can train this complex network architecture efficiently.

We build predictive models for foot-contact and foot-off events separately. Events in the output signal occur sparsely. That is, in *Y*_*r*_, the occurrence of a 1 (gait event) is rare compared to the occurrence of a 0 (non-event portion of the gait cycle). For this reason we need to balance the data or choose an objective function which accounts for the sample imbalance. Following machine learning literature [28], we choose to use a weighted binary cross-entropy, penalizing a misclassified 1 with 100: 1 proportion,

$$H(Y_{r},\hat{Y_{r}})=-\sum_{1\leq t\leq T_{r}}100\cdot Y_{r,t}\log{\hat{Y}}_{r,t}+\left(1-Y_{r,t}\right)\log\left(1-{\hat{Y}}_{r,t}\right),$$ (1)

where *Y*_*r*_ stands for the observed data and ${\hat{Y}}_{r}$ is the model prediction.

If we find *f* minimizing *H*(*Y*_*r*_, *f*(*X*_*r*_)), real-valued process *f*(*X*_*r*_) can be interpreted as the likelihood of an event. Peaks of sufficient magnitude in this process will correspond to predicted events.

For training the network we use sequences of fixed length *q* = 128 frames. Let *n*_0_ be an event in *Y*_*r*_. We use sequences [*n*_0_ − *Z*, *n*_0_ − *Z* + *q* − 1] where *Z* is a random integer sampled uniformly from {1, 2, …, *q*/2}. We apply this random shift *Z* in order to simulate starting from different points—to make the procedure more robust to various setups.

### 2.5 Evaluation framework

We use cross-entropy as a differentiable function that helps to identify the regions of interest. However, the practical usefulness of the method is better measured by an average number of frames between predicted annotations and the true annotations. Let *E*_*r*_ be the set of events and ${\hat{E}}_{r}$ the predicted set of events. Let *S* be a set of trials from the test set *T* on which numbers of predicted and the true annotations are equal.

1. coverage = |*S*|/|*T*|,
2. time = average time (measured by the number of frames) between true and predicted annotations on *S*, i.e. the minimum average absolute difference between pairs of values from *E*_*r*_ and ${\hat{E}}_{r}$.

Neither of these measures are smooth, so we cannot use them directly in the training. However, note that if we optimize the Eq (1) to 0, then our estimated ${\hat{Y}}_{r,t}$ matches exactly the true *Y*_*r*,*t*_ and therefore both coverage and time are also optimized.

### 2.6 Network architecture

Our pipeline can be described in the framework we presented earlier (Fig 1). First, we construct a neural network for approximating likelihood of an event. Second, we apply a peak detection algorithm for identifying the actual events.

**Fig 2:**
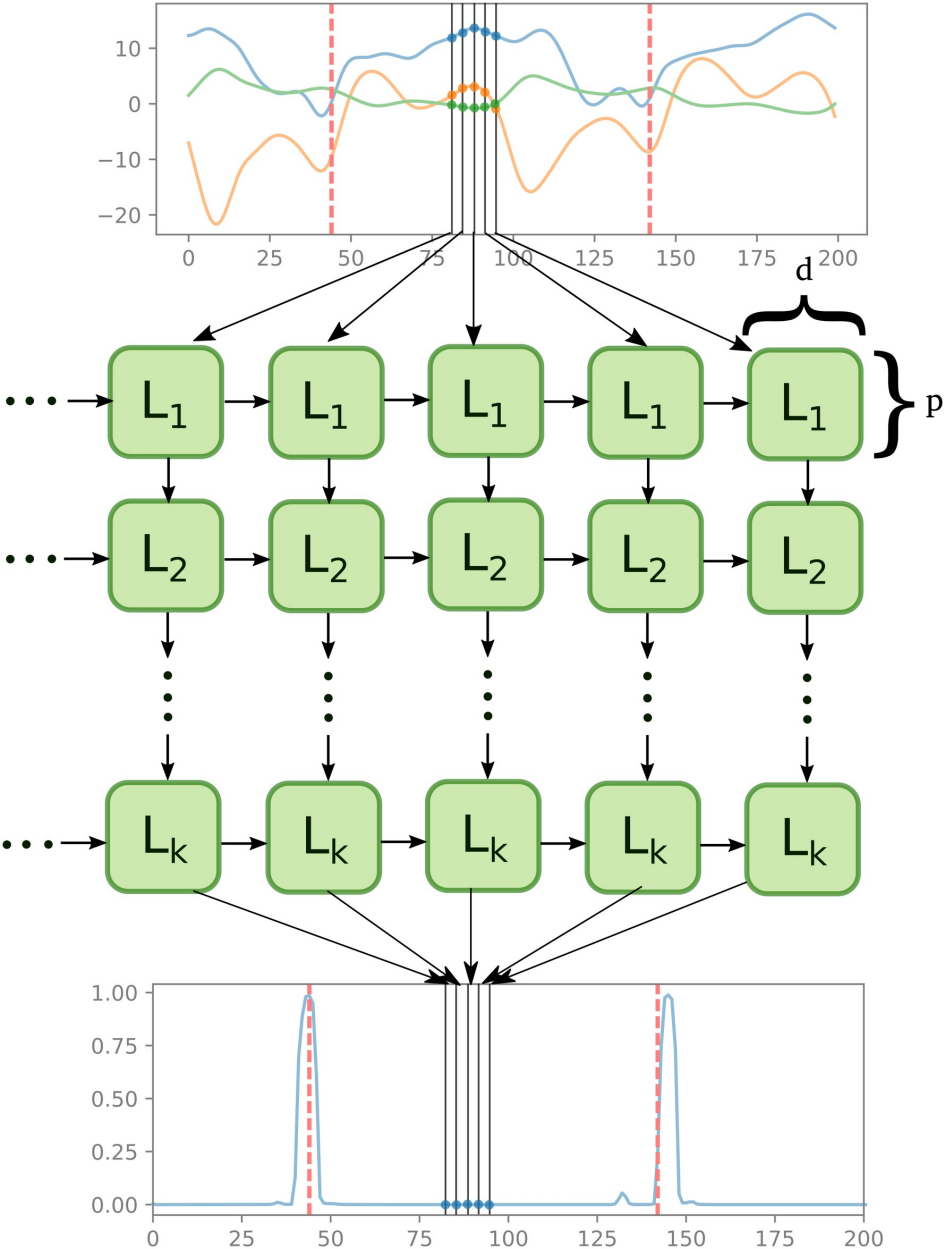
We approximate the mapping from the multivariate time series of kinematics and marker trajectories (top plot with only 3-dimensional time series for illustration) to the likelihood of an event (bottom plot) as a multi-layer neural network with LSTM layers (middle part of the figure). Each cell takes as input values from the previous time-step in the same level cell, and values from the current time-step in the higher level cell. Red dashed vertical lines represent the true event, and are used for training. Note that all LSTM cells *L*_*i*_ in every horizontal level are the same. Parameter *d* corresponds to the output dimension of each cell and *p* to the size of the hidden layer.

After a preliminary assessment of various parameter constellations, we test the following architectures of the network (Fig 2):

- *d* = 33 dimensional time series input (as described in Section 2.3),
- (*k* − 1) LSTM layers mapping the input to a *p*-dimensional time series,
- LSTM layer mapping the previous layer to a 2-dimensional time series,

where *k* ∈ {1, 2, 3} and *p* ∈ {16, 32, 64}.

We split our dataset into training (81%), validation (9%), and test (10%) sets. The validation set was selected randomly in each experiment. In the training phase we use cross-validation to choose the best combination of parameters (*k*, *p*) and the right set of features, where the best combination is chosen by comparing the predictions on the validation set. Once we establish the best combination, we retrain the network on the training and validation sets together and then compare our method with the two other algorithms using the test set. Due to the high correlation of within-subject trials, we split the dataset on the patient level.

### 2.7 Peak detection

We use an elementary peak detection algorithm, which defines a local maximum as a maximum point between two local minima [29]. More precisely let *X*(*t*) be a time series where *t* ∈ Ω = {1, 2, 3, …, *T*}, for some *T*. Let *δ* > 0 be some threshold for discriminating peaks. We call a point *t*_0_ a local maximum if and only if there exist points *t*_*l*_ ∈ Ω and *t*_*g*_ ∈ Ω such that: (i) *X*(*t*_*l*_) + *δ* < *X*(*t*_0_), (ii) *X*(*t*_*r*_) + *δ* < *X*(*t*_0_), and (iii) *X*(*t*_0_) is the maximimum point on the interval [*t*_*l*_, *t*_*r*_]. We define local minima analogically.

Points following the above definition can be found using an algorithm linear in time (*O(n)*), which scans through all the points one by one. For details on implementation and properties of this approach please refer to [30].

For our analysis, we normalize the outputs to [0, 1] interval and choose *δ* = 0.5. Our preliminary analysis indicated that the threshold *delta* has little effect on the results.

## 3 Experiments

### 3.1 Existing methods

We compare our method with two methods introduced in [14], coordinate-based and velocity-based algorithms. The coordinate-based method leverages the fact that at the foot-contact event, the distance in anterior-posterior direction between the heel and sacrum is maximized, whereas in the foot-off event the distance in anterior-posterior direction between the toe and the sacrum is minimized.

The velocity-based method uses the observation that at the foot-contact event the anterior-posterior component of the velocity of the heel marker changes from anterior to posterior. Similarly, the anterior-posterior component of the toe marker velocity changes from posterior to anterior at the foot-off event.

To ensure fair comparison between benchmark methods and our approach we attempted to maximize the performance of the benchmark. To this end, we used the following to techniques. First, in the peak detection algorithm described in Section 2.7 we tried different parameters *δ*. Second, we shifted all predictions by *k* number of frames ahead. Our analysis showed no sensitivity to parameter *δ*, while parameter *k* = 3 improves prediction in both algorithms. We used *δ* = 0.5 and *k* = 3 for the analysis.

While multiple other peak detection algorithms are available, none are commonly used in pathological gait. Comparison in [31] suggests that there is no clearly superior algorithm, and available algorithms yield comparable results.

## 4 Results

Our comparisons show a substantial advantage of our method over the two selected heuristic-based algorithms. Both coordinate-based and velocity-based methods fail to detect any events in between 7% and 16% of cases. In contrast, our algorithm fails to detect foot contact in less than 1% cases and foot-off in less than 5% cases. Manual investigation of these cases did not reveal any clear pattern distinguishing these cases.

**Fig 3:**
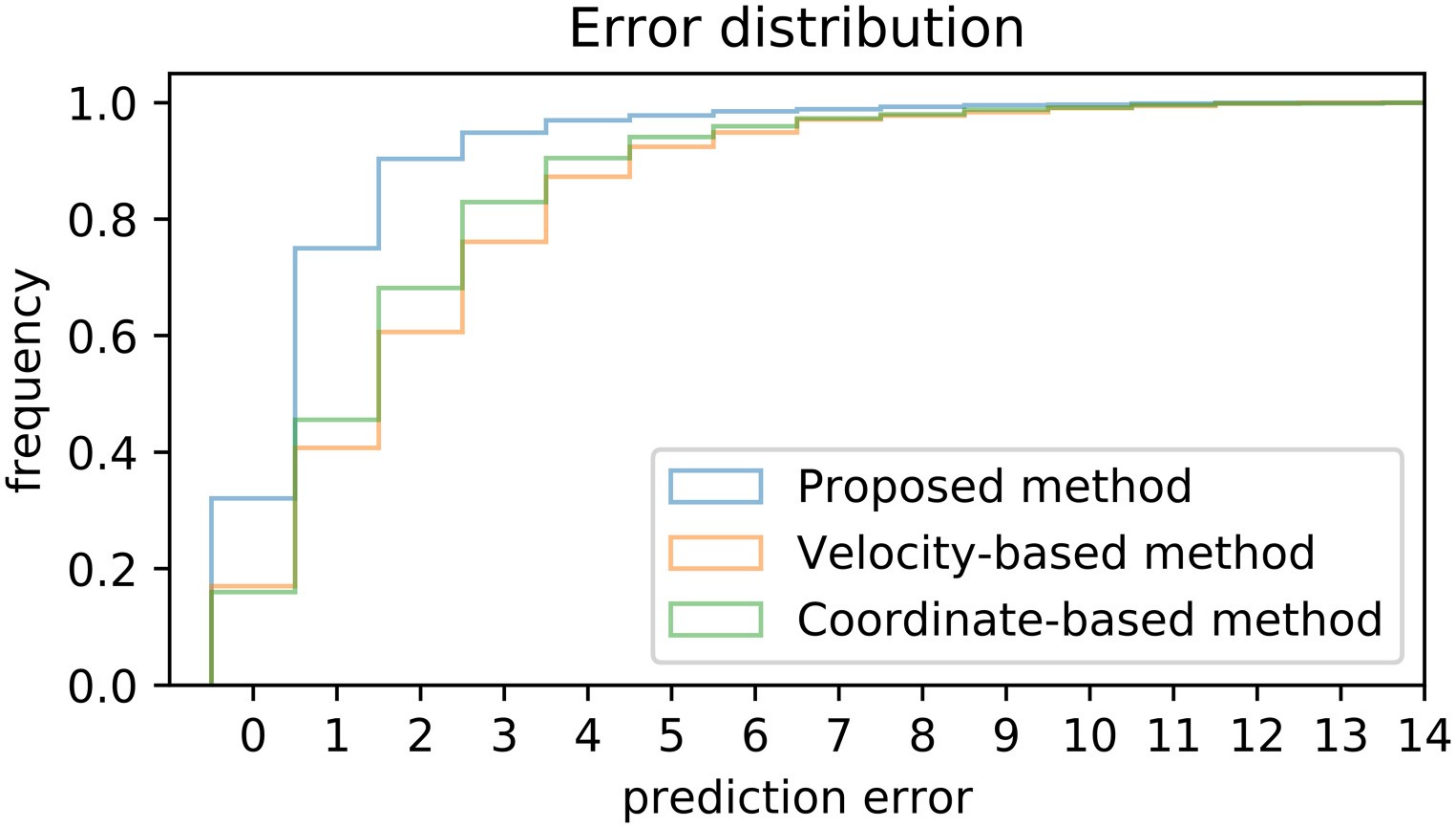
Comparison of sensitivity (true positive ratio) of three methods as a function of error tolerance (number of frames from the observed event within which we count the prediction as correct). Our method is off by at most 3 frames in 95% cases, whereas the coordinate-based algorithm only attains this level of accuracy in 83% cases.

Regarding the error in the cases where events were identified, our algorithm predicts the foot-contact within average 1.2 frames (∼10.0 ms) of the human expert identification (Table 2). This is a substantial improvement compared to 2.5 frames (∼20.8 ms) for the velocity-based algorithm, and 2.2 frames (∼18.3 ms) for the coordinate-based algorithm. Another important feature of the algorithm is the variability of the error, where the risk of an error greater than a given threshold is consistently and significantly lower for our algorithm compared to the velocity or coordinate algorithms (Fig 3).

**Table 2:** **Performance of the three algorithms on two measures on predicting foot-contact (FC) and foot-off (FO)**: Coverage corresponds to the frequency of detecting the event (larger is better), time is the average error in milliseconds from the ground truth as defined in Section 2.5 (smaller is better).

	coverage		time (in ms)	
	FC	FO	FC	FO
Coordinate-based	90%	84%	18.3	16.7
Velocity-based	93%	90%	20.8	18.3
Proposed method	**99%**	**95%**	**18.3**	**12.5**

Note that, the foot-off detection is consistently worse than the foot-contact detection. We expect that this is due factors commonly recognized by human annotators. The most likely such factor is that, for many children with severe neurological and orthopaedic impairments, there is no “clean” foot-off event, but rather the toe is gradually dragged off the floor. This results in an ambiguous event, corrupting both the human gold-standard and the algorithm estimation of the event. Further investigation is needed to determine sources of this effect.

## 5 Discussion

We combined three approaches to detect gait events: velocity-based, coordinate-based, and our machine learning approach derived from combining kinematic characteristics. We built a neural network leveraging a large dataset of the kinematics of children with cerebral palsy. Results indicate that the new model is superior to the heuristic-based models.

The model can reduce engineering costs and can allow more precise data processing. First, annotation of gait events can save around 30 minutes per trial, amounting to 150 hours of engineering time yearly in a clinic with only one patient visit per day. Second, thanks to this reduced cost, more data can be annotated. In many clinics, every trial consists of over 6 steps, but only 1 − 2 gait cycles are extracted; so over 60% of data is ignored for both clinical reporting and research. Third, as an automatic method, our algorithm is free from human errors and biases, which further contributes to the improvement of the work flow. Finally, accurate real-time event detection opens up intriguing possibilities including gait training, real-time feedback, tuning of electrical stimulation devices, or designing better assistive, orthotic, and prosthetic devices [32].

Results from [31], where three human annotators were asked to annotate events of 50 subjects, suggest that our algorithm surpasses human accuracy. In that study, annotations compared pair-wise are within two frames 84% of time and three frames 92% of time. Direct comparison using our algorithm on Bruening’s dataset would be necessary to prove superiority.

We release the model as part of this publication to stimulate further research and enable its use in practice. Our Python [33] implementation is based on the TensorFlow library [34]. It requires Biomechanics Toolkit [35] and runs on Linux (https://github.com/kidzik/event-detector). It can be used as-is, taking .c3d files as input and outputting annotated .c3d files. Moreover, relying on the principles of transfer learning [36], the trained network might be further tuned for a specific population using a much smaller training set.

While our method is directly applicable in practice for the data presented in this study, the training relies on a large dataset what can be constraining for wider adoption in specific clinical scenarios. Methods for decreasing both the sample size and the number of required features can further simplify our approach. First, expert-informed features, such as the ones used in heuristic-based approach, could greatly reduce the required data. Second, to reduce the required amount of data, one can apply principles of transfer learning [28] by tuning our model for a specific populations. This can be achieved by fixing the low-level features (the first layers of the network) and retraining only the last few layers.
